# Characterization and functional roles of paternal RNAs in 2–4 cell bovine embryos

**DOI:** 10.1038/s41598-019-55868-3

**Published:** 2019-12-30

**Authors:** Nicole Gross, Maria Giuseppina Strillacci, Francisco Peñagaricano, Hasan Khatib

**Affiliations:** 1 University of Wisconsin, Department of Animal Sciences, Madison, WI 53706 USA; 2 University of Milan, Department of Veterinary Medicine, Lodi, 26900 Italy; 3 University of Florida, Department of Animal Sciences, Gainesville, FL 32611 USA

**Keywords:** Agricultural genetics, Embryogenesis

## Abstract

Embryos utilize oocyte-donated RNAs until they become capable of producing RNAs through embryonic genome activation (EGA). The sperm’s influence over pre-EGA RNA content of embryos remains unknown. Recent studies have revealed that sperm donate non-genomic components upon fertilization. Thus, sperm may also contribute to RNA presence in pre-EGA embryos. The first objective of this study was to investigate whether male fertility status is associated with the RNAs present in the bovine embryo prior to EGA. A total of 65 RNAs were found to be differentially expressed between 2–4 cell bovine embryos derived from high and low fertility sires. Expression patterns were confirmed for protein phosphatase 1 regulatory subunit 36 (*PPP1R36)* and ataxin 2 like (*ATXN2L*) in three new biological replicates. The knockdown of *ATXN2L* led to a 22.9% increase in blastocyst development. The second objective of this study was to characterize the parental origin of RNAs present in pre-EGA embryos. Results revealed 472 sperm-derived RNAs, 2575 oocyte-derived RNAs, 2675 RNAs derived from both sperm and oocytes, and 663 embryo-exclusive RNAs. This study uncovers an association of male fertility with developmentally impactful RNAs in 2–4 cell embryos. This study also provides an initial characterization of paternally-contributed RNAs to pre-EGA embryos. Furthermore, a subset of 2–4 cell embryo-specific RNAs was identified.

## Introduction

Prior to EGA, the freshly fertilized zygote depends on maternal RNAs donated by the oocyte^1,2^. During EGA, the embryo degrades maternal RNAs and begins producing its own RNAs^1,2^. This process is termed the ‘maternal to zygotic transition’^1,2^. These oocyte RNAs are considered important precursors to successful embryonic development^1,3^. For instance, several maternal mRNAs alter cell fate. Depletion of the maternal mRNAs perilipin 2 (*PLIN2*)^4^, tripartite motif-containing 36 (*TRIM36*)^5^, and DND microRNA-mediated repression inhibitor 1 (*DND1*)^6^ disrupts the cortical rotation and microtubule formation events involved in axial patterning. Additionally, the presence of the maternal vegt protein (*VegT*) is instrumental for the determination of cell fate during primary germ layer formation in the blastula of *Xenopus* embryos^7^. Furthermore, proteins translated from the maternally-derived RNAs POU domain class 5 transcription factor 3 (*POU5F3*) and SRY-box transcription factor 3 (*SOX3*) mediate competence of cells prior to germ layer formation by remodeling chromatin structure just before initiation of inductive signaling in *Xenopus tropicalis* embryos^8^. The oocyte clearly influences embryonic development by contributing RNAs to the zygote at fertilization. However, sperm contributions to RNA patterns in the pre-EGA embryo are still unclear.

Older literature has suggested that the sperm only donates its chromosomes to the embryo at fertilization^9,10^. However, over time, studies have shown that the sperm contributes additional non-genetic components to the embryo^9,11^. It is now accepted that the sperm can transfer DNA methylation patterns^12,13^, mRNAs^14–18^, small non-coding RNAs^19^, and proteins^20,21^ to the embryo. Each of these non-genetic components is capable of regulating mRNA presence and activity^22–26^. Furthermore, sperm DNA methylation^27,28^, mRNAs^29^, small non-coding RNAs^30,31^, and proteins^32–34^ are all associated with male fertility status. The RNAs present in the embryo prior to EGA are important for determining cell fate and developmental success of embryos^4–8^. Previously, our lab reported that bull fertility status is associated with gene expression at the blastocyst stage^27^. However, the influence of male fertility over the mRNA content in pre-EGA embryos has not yet been evaluated on a whole-transcriptome scale.

Direct delivery of sperm RNA is perhaps the most straightforward influence of the sperm over pre-EGA embryo RNA content. Ostermeier *et al*.^14^ demonstrated that the sperm-specific transcripts protamine 2 (*PRM2*) and clusterin *(CLU*) could be transferred from human sperm to zona-free hamster oocytes. Further, they demonstrated that the transcripts were still present three hours post-fertilization^14^. A study in pigs confirmed that the paternal *PRM2* and *CLU* transcripts were passed to zygotes^16^. Additionally, studies have evaluated sperm transcript stability. The transcripts pregnancy specific beta-1-glycoprotein 1 (*PSG1*) and major histocompatibility complex, class I, E (*HLA-E*), but not *PRM2* were shown to remain stable for 24 hours following human sperm delivery to hamster oocytes^17^. Another group showed that the mouse sperm-derived forkhead box G1 (*FOXG1*) and Wnt family member 4 (*WNT4*) transcripts are transferred to the zygote^15^. Further, the *WNT4* transcript was translated at the 1-cell stage. The WNT4 protein remained stable following the loss of the transcript at the 2-cell stage^15^.

The functional importance of sperm-derived RNAs during embryonic development remains largely unknown. Sperm RNA function has been criticized because there is a large difference in RNA quantity between sperm and oocytes. A single spermatozoon contains 20–30 fg of RNA^35^, while a single oocyte contains 0.5 ng of RNA^36^. However, a small number of studies have demonstrated that sperm RNA function deserves a thorough investigation. In particular, the sperm-derived factor phospholipase C zeta (*PLCζ*) initiates the post-fertilization calcium oscillations that are required for embryo cleavage^20^. PLCζ is found as both a protein and an RNA in sperm^37^. *PLCζ* knockout male mice are infertile^38^. However, injecting *PLCζ* mRNA and the sperm of *PLCζ* knockouts into oocytes induces calcium oscillations and leads to the production of healthy pups^38^. The injection of only the mRNA extracted from sperm cells also leads to the production of calcium oscillations^39^. This could mean that the sperm-borne *PLCζ* RNA is translated prior to the activation of cell division^39^. Another example of a functional sperm RNA is DEAD-box helicase 3 Y-linked (*DDX3Y*). The sperm-borne *DDX3Y* transcript was found in freshly fertilized mouse zygotes, but not in oocytes^18^. Microinjection of an antisense RNA reduced the number of male cleavage-stage embryos produced and caused a lower cleavage rate of embryos^18^. These studies show that select sperm-borne RNAs may be indispensable during early embryonic development. Therefore, the milieu of paternally-contributed RNAs in the pre-EGA embryo should be further understood.

The first objective of this study was to evaluate whether the fertility status of bulls was associated with transcriptomic profiles of pre-EGA embryos. We utilized high-throughput sequencing to identify differentially expressed RNAs. Following validation, the differentially expressed RNA *ATXN2L* was knocked down in zygotes, as a proof of principle that paternally-contributed RNAs are important for development. The second objective of this study was to characterize the parental origin of the RNAs present in pre-EGA embryos on a whole-transcriptome scale. To do this, we integrated the pre-EGA embryo RNA-seq data with RNA-seq data from sperm and oocytes. This study provides new information about the paternal impact on pre-EGA embryo’s RNA content and function.

## Results

### Gene expression analysis of 2–4 cell embryos

A total of 65 genes were differentially expressed between embryos derived from high and low fertility sires. There were 35 genes with decreased expression and 30 genes with increased expression in the embryos from high fertility sires compared to the embryos from low fertility sires (FDR < 0.1). The subset of genes selected for validation were *ATXN2L*, *PPP1R36*, amyloid beta precursor protein binding family A member 1 (*APBA1*), plakophilin 2 (*PKP2*), cadherin EGF LAG seven-pass G-type receptor 3 (*CELSR3*), ELAV like RNA binding protein 4 (*ELAVL4*), WD repeat domain 93 (*WDR93*), Drebrin Like (*DBNL*), and ENSBTAG00000046943 (*LOC112444303 oncomodulin*). The genes *PPP1R36*, *PKP2*, *CELSR3*, and *ELAVL4* were chosen because they had previously-identified associations with reproductive development and fertility. On the other hand, *ATXN2L*, *APBA1*, *WDR93*, *DBNL*, and *LOC112444303 oncomodulin* were selected because their roles in fertility were still unclear. In total, nine genes were chosen for qRT-PCR validation (Table 1). All three validation replicates for *ATXN2L* and *PPP1R36* reproduced the same trend found in RNA-Seq results. The gene *ATXN2L* had an average fold difference of 2.45 (*P* = 0.052), and *PPP1R36* had an average fold difference of 4.08 (*P* = 0.065). The genes *WDR93*, *ELAVL4*, and *LOC112444303 oncomodulin* showed replication of RNA-Seq results for two out of three biological validation replicates. *WDR93* had an average fold difference of 0.65 (*P* = 0.39), *ELAVL4* had an average fold difference of 0.43 (*P* = 0.13), and *LOC112444303 oncomodulin* had an average fold difference of 0.70 (*P* = 0.40). The genes *DBNL*, *CELSR3*, *APBA1*, and *PKP2* were not consistent across biological validation replicates. The 65 differentially expressed genes are found in Supplementary Table S1. Additionally, there was no significant difference in the cleavage rate between high and low fertility sire embryos (*P* = 0.70) (Supplementary Table S2).

**Table 1:** qRT-PCR results for nine genes identified as differentially-expressed between high and low fertility sires.

Gene	Average Fold Change	Standard Deviation	Number of Validated Replicates (>1.5-fold)	P-value
ATXN2L	2.45	0.79	3	0.05
DBNL	2.84	3.01	1	0.44
WDR93	0.65	0.5	2	0.39
ELAVL4	0.43	0.32	2	0.13
PPP1R36	4.08	2.23	3	0.06
CELSR3	NA (Ct > 33)	NA (Ct > 33)	1	NA (Ct > 33)
LOC112444303 oncomodulin	0.7	0.64	2	0.4
APBA1	1.16	1.12	1	0.8
PKP2	1.24	0.71	1	0.84

### Knockdown of ATXN2L with gapmer treatment

To test the possible role of *ATXN2L* in embryo development, knockdown experiments were performed using gapmer technology. Embryos which were treated with *ATXN2L* gapmer at the zygote stage showed a 22.9% increase in blastocyst rate compared to control embryos (P < 0.05). The fold change in *ATXN2L* was 0.44 in treated vs. control embryos (P < 0.05).

### Origin of RNAs

**Figure 1:**
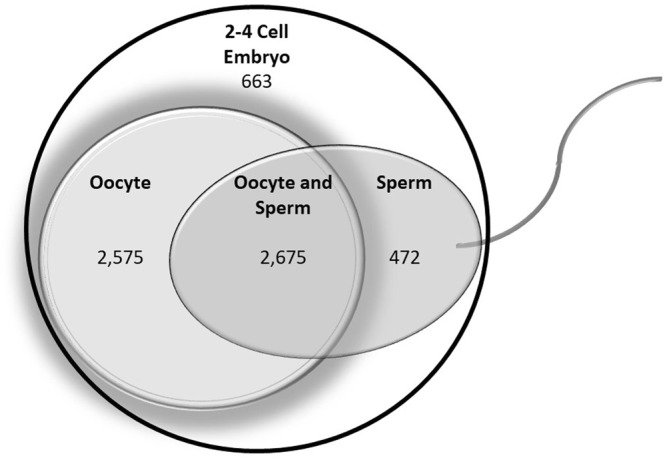
Parental Origin of 2–4 Cell Embryo RNAs. The Venn diagram displays the origin of the RNAs present in 2–4 cell embryos. Detailed gene names are provided in Supplementary Table S3.

The parental origin of 6385 RNAs from 2–4 cell embryos was characterized (Fig. 1). There were 2675 RNAs derived from both the sperm and the oocyte. An additional 2575 RNAs were exclusively oocyte-derived, while 472 RNAs were exclusively sperm-derived (Supplementary Table S3). Finally, there were also 663 RNAs that were found only in 2–4 cell embryos.

**Figure 2:**
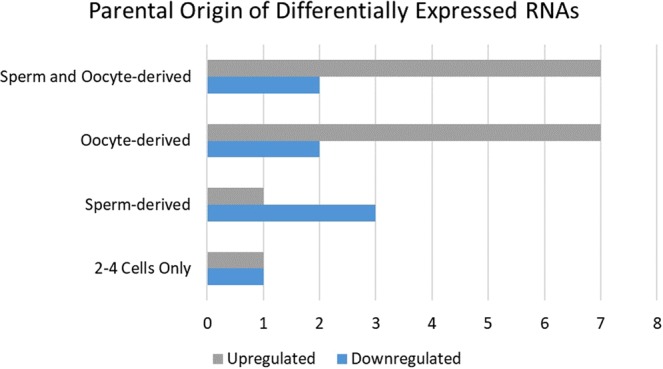
Parental Origin of 24 Differentially Expressed RNAs between high- and low-fertility sires. The parental origin of an additional 41 genes was considered provisional and can be found in Supplementary Table S3.

The expression levels of 24 of the differentially expressed RNAs (FDR < 0.1) in the 2–4 cell embryos was further determined. Of these, eight RNAs were downregulated and 16 RNAs were upregulated in embryos from high fertility sires versus low fertility sires. A total of four differentially expressed genes were sperm-derived. There were also two differentially expressed genes found exclusively in 2–4 cell embryos and nine that were oocyte-derived. Additionally, the sperm and oocyte co-contributed nine differentially expressed genes (Fig. 2). The parental origins of the remaining 41 differentially expressed genes were considered provisional and can be found in Supplementary Table S2.

## Discussion

The RNAs donated by the oocyte at fertilization are known to affect the developmental competence of embryos^4–8^. It is also recognized that the sperm donates a variety of non-genetic elements^12–21^ during fertilization that can alter RNA expression^22–26^. This study demonstrates that male fertility is associated with RNA content in the pre-EGA embryo. The functionality of one of these RNAs was interrogated through knockdown experiments. Additionally, this study reveals a substantial number of RNAs that are directly contributed from the sperm to the embryo at fertilization. Furthermore, a suite of transcripts that are specific to the 2–4 cell embryo have also been identified.

Several of the differentially expressed genes identified through this study have been previously associated with fertility. For example, a genome-wide association study found that *PKP2* and CTTNBP2NL N-terminal like (*CTTNBP2NL*) are associated with conception rates in Holstein cows^40^. Knockdown of *PKP2* and overexpression of *CTTNBP2NL* each resulted in reduced embryo implantation rates in mice^40^. In this study, *PKP2* was upregulated and *CTTNBP2NL* was downregulated in embryos from high fertility sires. Additionally, both genes were expressed in sperm. Therefore, this study demonstrates that there is paternal control over the presence of these RNAs in pre-EGA embryos. Further, the direction of differential expression for these RNAs in the pre-EGA embryos reinforces previous findings about their functions in fertility.

Additional differentially expressed genes have been associated with the regulation of spermatogenesis. For instance, the gene *PPP1R36* drives autophagy during spermatogenesis^41^. Inhibiting autophagy in mice leads to infertile spermatozoa^42^. *PPP1R36* was found in spermatozoa in this study. Additionally, *PPP1R36* was upregulated in embryos from high fertility sires. Sperm-specific knockout mice for the autophagy regulator autophagy related 5 (*ATG5*) generate embryos that fail to develop beyond the 4–8 cell stage^43^. Thus, future studies should evaluate whether *PPP1R36* is transferred to the zygote to help regulate autophagy. The mechanistic target of rapamycin kinase (*mTOR*) is another spermatogenesis-regulating gene that was differentially expressed in pre-EGA embryos. *mTOR* can shape male reproductive potential by affecting spermatogonial stem cell maintenance, Sertoli cell physiology, and blood-testis barrier architecture^44^. Blocking *mTOR* activity in mouse Sertoli cells is detrimental to sperm quality and causes infertility^45^. *mTOR* was upregulated in embryos of high fertility sires, and was also found in sperm. Future studies should evaluate whether these genes serve a dual purpose in regulating both spermatogenesis and early embryonic development.

Although many of the differentially expressed genes in this study were sperm-derived, there was a subset of these genes that were either oocyte-derived or embryo-specific. Therefore, alternative mechanisms for paternal regulation of pre-EGA RNA may be possible. Previously, our lab showed that differential DNA methylation patterns in sperm from high and low fertility sires was correlated with altered expression of the same genes in blastocysts from high and low fertility sires^27^. In this study, the paternally-expressed imprinted genes *APBA1* and GATA binding protein 3 (*GATA3*)^46^ were both upregulated in embryos from high fertility sires. Altered DNA methylation in sperm of these genes is associated with motility^47^ and male fertility status^48^, respectively. *APBA1* transcripts were found in sperm, but *GATA3* was categorized as oocyte-derived in this study. Future studies should evaluate alternative sperm-derived contributions that may regulate pre-EGA embryo RNA, such as DNA methylation.

The knockdown of *ATXN2L* led to an increase in blastocyst development. Intriguingly, the expression of this gene was elevated in 2–4 cell embryos from high fertility sires. However, *ATXN2L* can have equally dynamic effects when its expression is increased or decreased^49^. *ATXN2L* is considered a regulator of stress granule and processing body (P-body) formation^49^. When *ATXN2L* is upregulated in HeLa cells, P-body numbers are reduced and stress granule numbers are increased^49^. Conversely, downregulation of *ATXN2L* in HeLa cells causes a reduction of stress granules^49^. P-bodies are present in non-stressed cells and contain non-translating mRNA and proteins which facilitate mRNA decay and translational repression^50^. Stress granules are formed when blocks of translation initiation occur in cells, and they contain various translation initiation factors and regulators of mRNA stability^50^. Control over RNA presence and activity is crucial to EGA^51^. Therefore, *ATXN2L* may require a careful balance during early embryonic development. Previously, a “maternal mode” and a “zygotic mode” have been proposed to initiate mRNA decay during EGA^51^. In the future, the use of *ATXN2L* as a “paternal mode” to regulate the mRNA presence should be explored. It is unclear whether the knockdown of *ATXN2L* is truly beneficial for embryonic development. However, our results indicate that reducing the expression of this gene can upregulate blastocyst development. Future studies should investigate the role of *ATXN2L* when it is overexpressed at the zygote stage. Moreover, it will be important to elucidate its possible role in regulating maternal RNAs. *ATXN2L* was not proven to be directly contributed by sperm in this study. Therefore, the paternal mechanism of control over *ATXN2L* expression should also be further assessed.

Delivery of individual sperm RNAs has been demonstrated in a variety of species^14–18^. The *CLU* RNA was shown to be transferred from sperm to oocytes in multiple studies^14,16^. *CLU* was found in sperm, oocytes, and 2–4 cell embryos in our dataset. This indicates it is both maternally and paternally-contributed in bovine. Additionally, calcium binding tyrosine phosphorylation regulated (*CABYR*) was previously identified as a sperm-specific RNA delivered to the zygote in mice^52^. Our study also confirmed this finding. The full subset of sperm-derived RNAs will provide a useful reference to identify fertility-related interactions between paternally-derived RNAs. For instance, this study revealed that a binding partner of *CABYR* called A-kinase anchoring protein 3 (*AKAP3*) was also sperm-derived. CABYR, AKAP3, and Ropporin form a complex in the fibrous sheath of sperm, which has been implied to function as signaling for capacitation^53^. Additional research should be carried out to determine whether these RNAs produce proteins that form functional complexes during early embryonic development.

Ultimately, these data also allowed additional insight into the minor EGA of the 2–4 cell embryo. Minor EGA is a pre-emptive wave of transcriptional activity which occurs at the 1 or 2–4 cell stage of bovine embryo development^54^. This study uniquely compiles information from both the maternally and paternally contributed RNAs. Therefore, we identified a novel subset of RNAs that are exclusively transcribed by the 2–4 cell bovine embryo during minor EGA. Conversely, a subset of the gene transcripts we identified as paternally-derived in this study is actively transcribed the 2–4 cell embryo. For example, we classified cyclin A2 (*CCNA2*) and cyclin dependent kinase 2 (*CDK2*) as maternally-derived RNAs in our study. These genes cooperate during activation of the embryonic genome^55^. In another study, Graf *et al*.^49^ found that *CCNA2* and *CDK2* belong to the category of RNAs that are maternally-derived but also transcribed by the embryo. However, several RNAs that were identified as embryo-specific through this study were also found to be present at the 4-cell stage in the study by Graf *et al*.^49^. These were deoxyribonuclease 1 (*DNASE1*), membrane associated ring-CH-type finger 3 (*MARCH3*), and ras-related C3 botulinum toxin substrate 3 (*RAC3*). DNASE1 can regulate DNA turnover and affects the stability of promoters^56^. MARCH3 mediates protein ubiquitination of Fc gamma receptor (FcγR), which affects responses to antibody-coated tumor cells^57^. RAC3 is a GTPase that alters cell growth by promoting cell adhesion and spreading^58^. These genes represent typical functions for early EGA RNAs transcribed by the embryo. However, this study also uncovered less traditional functions in the novel subset of early EGA genes.

Many of the unique embryo-specific RNAs in this study have roles in cell-cell signaling. These RNAs may serve as important regulators of embryo-mother communication during preimplantation development. For example, this study identified that sex hormone binding globulin (*SHBG*) expression was embryo-specific. SHBG is an androgen and estrogen transport protein which regulates plasma concentration of steroid hormones^59^. A deleterious mutation in *SHBG* is associated with prenatal death in dairy cattle^60^. This protein can be internalized by neurons or prostate cancer cells^61,62^. Further, SHBG can transport sex steroids into these target cells^61,62^. Another transcript found only in 2–4 cell embryos was tumor necrosis factor (*TNF*), a known mediator of embryo-maternal communication in mammals^63^. This gene modulates prostaglandin F2alpha (PGF2α) and is thought to aid in transferring the embryo from the oviduct to the uterus^64^. Additionally, secreted seminal-vesicle Ly-6 protein 1 (*SOLD1*) expression was also found to be embryo-specific in this study. SOLD1 is a signal at the fetomaternal interface that can regulate trophoblast invasiveness^65^. Future studies should interrogate whether these 2–4 cell embryo-specific RNAs can regulate signaling in the reproductive tract.

In conclusion, the present study demonstrated that sperm assists in regulating a substantial amount of pre-EGA RNA content in the early embryo and that male fertility is associated with RNA patterns in pre-EGA embryos. The knockdown of one of these genes, *ATXN2L*, resulted in substantially increased blastocyst development. This study also provided a whole-transcriptome characterization for oocyte- and sperm-contributed RNAs prior to EGA. A novel group of RNAs that are produced specifically by the 2–4 cell embryo was also identified. Future studies should evaluate whether non-RNA components contributed by the sperm to the zygote are capable of regulating pre-EGA RNA content in the embryo. Additionally, the roles of paternally-influenced RNAs in pre-EGA embryos should be further explored.

## Methods

### Ethics statement

This study did not require approval from the Animal Care and Use Committee. Cows used for oocyte aspirations were not cared for at the University of Wisconsin – Madison facilities. Ovaries used for oocyte aspirations were purchased from Applied Reproductive Technology, LLC (Monona, WI, USA), and we were permitted by the company to perform *in vitro* fertilization (IVF) using the ovaries.

### Bull semen selection

Semen samples were donated by Semex, Canada and the fertility status of sires was designated by the company as either high or low fertility based on the Repromax™ system, which simultaneously accounts for Sire Conception Rate (SCR), Agri-Tech Analysis (ATA), and Canada’s Non-Return Rate (NRR) data. Permission was granted by Semex to perform IVF. A total of 12 bulls were used for embryo production, which included six high fertility and six low fertility bulls.

### *In vitro* embryo production

**Figure 3:**
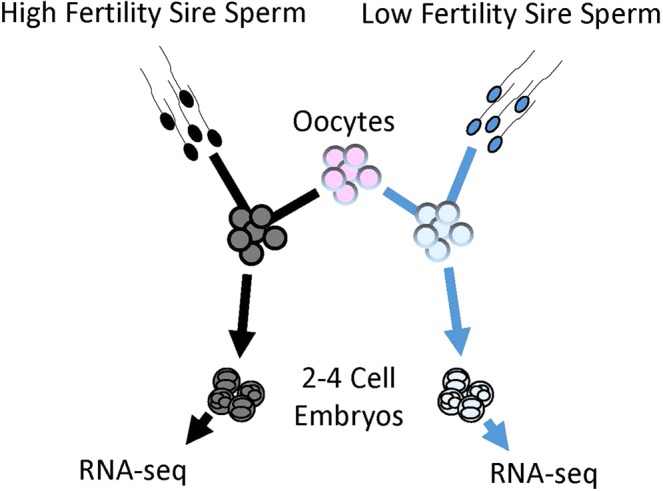
Schematic of experimental design for embryo generation. Each replicate included one high fertility and one low fertility sire.

*In vitro* production of embryos was performed as described previously^66,67^. Ovaries were supplied by Applied Reproductive Technology, LLC (Monona, WI, USA), from which follicles were aspirated for oocyte collection. Oocytes were washed in a TL-Hepes solution which contained 3% bovine serum albumin, sodium pyruvate, and gentamicin. Cohorts of 10 oocytes were matured for 24 h in 50 µl of M-199 maturation media which contained gonadotropins (FSH and LH), estradiol, sodium pyruvate, 10% fetal bovine serum, and gentamicin. In each of the six IVF replicates, matured oocytes were washed in the supplemented TL-Hepes solution and then split evenly into two groups for fertilization (200–380 oocytes/sire). One oocyte group was fertilized using a high fertility sire, and the second group was fertilized using a low fertility sire (Fig. 3). Overall, a total of 1737 oocytes were allocated to high fertility bulls and 1606 oocytes were allocated to low fertility bulls across six IVF replicates. New bulls were used in each replicate. In total, six high fertility bulls and six low fertility bulls were used for 2–4 cell embryo generation. Age differences of the bulls paired in each IVF replicate were checked using a paired t-test, and bull pairs used for IVF showed no substantial differences in age. Cohorts of 10 oocytes were transferred to 44 µl drops of fertilization medium, which was supplemented with fatty acid-free bovine serum albumin (FAF-BSA), sodium pyruvate, and gentamicin. Sperm preparation was performed with a Percoll gradient as previously described^68^. The final sperm concentration was adjusted to 1 million per mL, using 2 µl of sperm per drop. Penicillamine-hypotaurine-epinephrine and heparin (2 µl each) were added to fertilization droplets. Oocytes and sperm were co-cultured for 20 h. Then, presumptive zygotes were stripped of cumulus cells, washed with the supplemented TL-Hepes solution, and placed in 50 µl drops of CR1aa culture media^69,70^ which were supplemented with FAF-BSA, sodium pyruvate, amino acids, and gentamicin. Presumptive zygotes were cultured in cohorts of 20–25. At 40 h post-fertilization, embryos that had reached the 2–4 cell stage were counted and collected into 100 µl of lysis solution for RNA-Sequencing (RNA-Seq). Statistical analysis of cleavage rates was performed using a paired t-test. Three biological replicates of embryo groups containing 37–70 cleavage-stage embryos for each high and low fertility bull were used for RNA-Seq.

### RNA-Seq profiling of 2–4 cell embryos and statistical analysis

Total RNA was extracted from embryo groups using the RNAqueous™-Micro Total RNA Isolation Kit (Ambion, Austin, TX, USA). The Repli-G WTA single cell kit (Qiagen, Germantown, MD, USA) was then used for whole transcriptome amplification (WTA). Purification of cDNA was carried out using Agencourt AMPure XP (Beckman Coulter, Brea, CA, USA). Library preparation was performed using the Nextera DNA library preparation kit (Illumina, San Diego, CA, USA). Then, 150 bp paired-end sequencing was carried out on the HiSeq X Ten (Illumina). The quality of fastq files containing the raw sequence reads was evaluated using FastQC software (v 0.11.8). Adapters were trimmed from all sample fastq files using Trimmomatic software (v. 0.38) and the reads were simultaneously quality trimmed by removing reads shorter than 35 bp. All the trimmed files were re-checked for quality using FastQC software. Paired-end read files were aligned to the UMD3.1 bovine genome assembly (Bos taurusUMD3.1.dna.toplevel.fa) using Tophat (v 2.1.1)^71^. The number of reads per gene in each sample was calculated by HTSeq-count (v 0.6.1)^72^ using UMD3.1 as reference genome (Bos_taurus.UMD3.1.94.gtf). Differential expression analysis was performed on 2–4 cell samples using EdgeR (v 3.20.7). The statistical tests were corrected for multiple testing using the Benjamini-Hochberg method as implemented in EdgeR^73^. A false discovery rate (FDR) cutoff of 10% was used to identify significant genes.

### Evaluation of gene expression in biological replicates

Three additional biological replicates of *in vitro*-produced 2–4 cell embryos were generated for validation purposes. New high- and low-fertility sires were used for each replicate. RNA was extracted from groups of 180–280 2–4 cell embryos for each sire. Whole transcriptome amplification was performed as described above. The iTaq Universal SYBR Green Supermix (Bio-Rad) was used for quantitative real-time PCR (qRT-PCR). Cycling was performed with a CFX-Connect Real-Time PCR Detection System (Bio-Rad), under the following conditions: 95 °C for 30 seconds, followed by 40 cycles of 95 °C for 5 seconds, and 60 °C for 30 seconds. To select a reference gene, the genes beta-actin (*ACTB*), ATP synthase F1 subunit beta (*ATP5B*), beta-2-microglobulin (*B2M*), and calnexin (*CANX*) were compared for expression stability across 2–4 cell embryo samples. The most stable gene, *ACTB*, was selected as the internal control^74^. All primers were designed to span an intron, in order to avoid DNA amplification (Supplementary Table S4). Fold change in gene expression was determined using the 2^−ΔΔ^CT method as described^75^. If a sample CT was greater than 33, it was considered beyond the threshold for quantification. Validation within individual biological replicates was considered, and those replicates with >1.5-fold difference in accordance with sequencing results were categorized as individually validated. For determination of significance across all three replicates, a paired t-test of normalized gene expression values (ΔCT) was used.

### Knockdown of *ATXN2L* using gapmer supplementation

*ATXN2L* was selected for further functional analysis based on its significance in validation, and because its role in fertility had not yet been explored. Knockdown was performed with a locked nucleic acid gapmer specific to *ATXN2L*, which was custom designed by Qiagen. Both 1 µM and 3 µM concentrations of the gapmer were evaluated, and the optimal concentration of 1 µM was selected. Each gapmer was supplemented to culture media 24 hours post-fertilization. Simultaneously, a control group of embryos was cultured, where an equivalent volume of water (vehicle of gapmer) was supplemented. Embryos remained in culture until blastocysts were graded at eight days post-fertilization. For grading, embryos were categorized as either blastocyst or degenerate. The blastocyst rate was calculated as the percentage of cleaved embryos that developed to the blastocyst stage. Three IVF replicates of *ATXN2L* gapmer knockdowns were carried out. Additionally, a cohort of embryos was supplemented with a bovine negative control gapmer, which is a scramble sequence designed so that it does not align to any known bovine sequences. Immediately after blastocyst grading, embryos were collected in lysis buffer, and RNA was extracted with the same method described above for the 2–4 cell embryos. Gene expression analysis was performed in order to confirm gapmer activity, and fold change in gene expression was calculated using the 2^−ΔΔCT^ method^75^. The significance of both blastocyst rate and gene expression was determined using a paired t-test.

### Identification of RNAs expressed in sperm

Sperm RNA was extracted from the two sires (one high fertility, one low) from one of the sequenced IVF replicates. One straw of cryopreserved semen per sire was subjected to RNA extraction. Each straw was thawed for one minute at 35–37 °C. Thawed semen was transferred to a 1.5 mL tube and centrifuged for four minutes at 4000 RPM. The supernatant was removed, and cells were suspended in a somatic cell lysis buffer^76^ for four minutes on ice. Sperm was then centrifuged four minutes at 4000 RPM and lysis supernatant was aspirated. Somatic cell lysis buffer suspension was repeated once. Following somatic cell lysis, sperm were suspended in 0.75 mL of TRIzol (Invitrogen, Carlsbad, CA, USA), and RNA extraction was carried out according to manufacturer instructions. Then, RNA was amplified through WTA, purified, and sequenced. Reads were processed as described for 2–4 cell embryo samples. RNAs with a read count greater than five in either sample were considered to be expressed in sperm.

### Identification of RNAs expressed in oocytes

Studies that evaluated bovine oocyte RNA expression in multiple replicates were identified using NCBI Gene Expression Omnibus (GEO) (http://www.ncbi.nlm.nih.gov/geo). Two studies provided freely available data on bovine oocyte RNA expression in multiple replicates^77,78^, and were thus used for comparison to our sequencing results. Data from Xie *et al*.^77^ is under accession number GSE18290 and data from Graf *et al*.^49^ is under accession number GSE52415. The averages across oocyte replicates was used for comparison. The data from Xie *et al*.^77^ was generated using the Affymetrix GeneChip® Bovine Genome Array, which includes all high quality and publicly identified bovine transcripts. The Affymetrix-gene-code was converted to gene ID through DAVID^79,80^, and a minimum probe threshold of 40 raw intensity units^81^ was used as a cutoff to classify whether or not a gene was expressed. The data from Graf *et al*.^49^ were generated through RNA-Seq and aligned to UMD 3.1, and a cutoff of greater than five reads^78^ was used to classify whether a gene was expressed in oocytes for this dataset.

### Characterization of RNA origin

To classify the parental origin of RNAs, the reads from 2–4 cell embryo samples were merged with sequencing results from sperm samples and literature-derived oocyte RNA expression data. Any gene with greater than five reads in at least one of the six replicates of 2–4 cell embryos was considered expressed, and the thresholds stated above were used to determine sperm and oocyte expression. Genes that did not meet the threshold for any of the 2–4 cell embryo samples were omitted from the analysis. The parental origin of RNAs was determined based on RNA expression in different sample types. Ultimately, the RNAs were sorted into four categories: (1) both oocyte- and sperm-derived (2) oocyte-derived; (3) sperm-derived; (4) only 2–4 cell embryo. If a gene was only found in one gamete-specific dataset (rather than both) it was labeled as provisional. Provisionally-labeled genes were not included in final counts of parental origin, but all genes and parental origin labels can be found in Supplementary Table S3.

### Accession codes

Oocyte RNA expression data from Xie *et al*.^77^ is under accession number GSE18290, and oocyte RNA expression data from Graf *et al*.^49^ is under accession number GSE52415.

## Data availability

The majority of data generated is published in the current study (and its Supplementary Information files). The datasets generated during the current study are available from the corresponding author on reasonable request.

## Supplementary information

Supplementary Table S1

Supplementary Table S2

Supplementary Table S3

Supplementary Table S4
